# The Utility of Artificial Intelligence in the Diagnosis and Management of Pancreatic Cancer

**DOI:** 10.7759/cureus.49560

**Published:** 2023-11-28

**Authors:** Vikash Kumar, Mrunanjali Gaddam, Amr Moustafa, Rabia Iqbal, Dhir Gala, Mili Shah, Vijay Reddy Gayam, Praneeth Bandaru, Madhavi Reddy, Vinaya Gadaputi

**Affiliations:** 1 Internal Medicine, The Brooklyn Hospital Center, Brooklyn, USA; 2 Internal Medicine, American University of the Caribbean School of Medicine, Sint Maarten, SXM; 3 Gastroenterology and Hepatology, The Brooklyn Hospital Center, Brooklyn, USA; 4 Gastroenterology and Hepatology, Blanchard Valley Health System, Findlay, USA

**Keywords:** deep learning, screening, machine learning, artificial intelligence, pancreatic cancer

## Abstract

Artificial intelligence (AI) has made significant advancements in the medical domain in recent years. AI, an expansive field comprising Machine Learning (ML) and, within it, Deep Learning (DL), seeks to emulate the intricate operations of the human brain. It examines vast amounts of data and plays a crucial role in decision-making, overcoming limitations related to human evaluation. DL utilizes complex algorithms to analyze data. ML and DL are subsets of AI that utilize hard statistical techniques that help machines consistently improve at tasks with experience. Pancreatic cancer is more common in developed countries and is one of the leading causes of cancer-related mortality worldwide. Managing pancreatic cancer remains a challenge despite significant advancements in diagnosis and treatment. AI has secured an almost ubiquitous presence in the field of oncological workup and management, especially in gastroenterology malignancies. AI is particularly useful for various investigations of pancreatic carcinoma because it has specific radiological features that enable diagnostic procedures without the requirement of a histological study. However, interpreting and evaluating resulting images is not always simple since images vary as the disease progresses. Secondly, a number of factors may impact prognosis and response to the treatment process. Currently, AI models have been created for diagnosing, grading, staging, and predicting prognosis and treatment response. This review presents the most up-to-date knowledge on the use of AI in the diagnosis and treatment of pancreatic carcinoma.

## Introduction and background

Pancreatic carcinoma (PC) is a lethal cancer of the pancreas. The most prevalent type of pancreatic cancer is pancreatic ductal adenocarcinoma (PDAC). It should be considered that the majority of cases are PDAC when the broad term “pancreatic cancer” is used. PDAC is categorized as one of the leading causes of cancer mortality worldwide. Unfortunately, pancreatic cancer usually shows few or no symptoms until it has either locally advanced or metastatically spread leading to poor survival rates and posing a significant diagnostic challenge. Due to close proximity to the major blood vessels, the tumor can easily invade them [1], making 80-85% of tumors non-resectable at the time of diagnosis [2]. Therefore, in addition to addressing the risk factors associated with it such as smoking, alcohol, chronic pancreatitis, diabetes, obesity, and *Helicobacter pylori *infection, appropriate timely diagnosis and treatment also improve survival rates. According to a multicenter study, individuals with PC identified through screening had a median survival time of 9.8 years as opposed to 1.5 years for patients with PC identified with non-screening.

Generally, CT scan with contrast media enhancement and magnetic resonance imaging (MRI) are considered for the diagnosis/staging of pancreatic cancer. However, Endoscopic Ultrasound (EUS) has a higher sensitivity for identifying solid pancreatic lesions less than 2 cm when compared to CT and MRI [3]. A biopsy is the gold standard for diagnosing pancreatic cancer. Even the best-validated PC biomarker, carbohydrate antigen 19-9 (CA19-9), does not have enough accuracy and specificity in screening early PC, and a lack of biomarker in early PC make it challenging to diagnose at early staging. As a result, numerous researchers are still striving to develop new early screening methods for PCs. As the images vary with disease progression, it is not always simple to interpret and evaluate the resulting images. There can also be interobserver variability in reading radiographic images, which can be improved with the use of AI. Similarly, several factors may have an influence on prognostic factors and treatment response.

Artificial intelligence (AI) is the computer simulation of the human intelligence process. The concept of AI emerged in the 1950s, but real progress was made only a few years ago [4]. The utilization of AI is rapidly expanding and is increasingly useful in understanding gastrointestinal diseases [5-7]. AI acquires a flexible structure by employing mathematical formulas and fundamental statistical concepts. As it is trained with real-world data in a particular domain, it can infer and reason. 

One widely used subset of AI is machine learning (ML). ML examines data to develop algorithms that can recognize distinct behavior forms and confirm predictive models. ML focuses on developing models that assist machines in making predictions or judgments without explicit programming. Various ML techniques, for instance, support vector machines (SVM), artificial neural networks (ANNs), classification, and regression trees, seem to be employed in various investigations in the medical discipline [4]. New algorithms are seemingly introduced every day. Some show the potential to have a major impact on image interpretation.

Another AI technique called Deep learning (DL) has become a promising tool for image analysis in medicine [8-11]. DL is a part of the ML technique that uses deep multilayered neural network algorithms to make predictions based on discrete inputs. The model uses an iterative process to take output predictions and reanalyze the model to further improve accuracy, continually improving with each new data point. DL has an edge over other conventional ML techniques as it uses “representation learning,” a technique by which the AI model recognizes relationships within a data set that human operators cannot notice [11].

In summary, ML is a core component of AI, and to implement it, DL is used. ML and DL have been successful at predicting the risk of gastric cancer [12].

## Review

Artificial intelligence in the diagnosis of pancreatic cancer

As the medical field and knowledge continue to expand, AI has gained importance due to its efficiency in processing and organizing data. The use of AI has been bringing new tools that have been helping in early diagnosis, risk stratification, and improving outcomes in patients with PC [13].

With the advancement in the field of AI, many tools are available that help to measure biomarkers and analyze complex features and tissue characteristics.

For the diagnosis of PC, imaging using CT, EUS, MRI, and positron emission tomography (PET) scan is required, which requires an expert radiologist to interpret the image. Previously used modalities and techniques are summarized in Table 1.

**Table 1:** Summary of the advantages and disadvantages of the diagnostic modalities used to diagnose pancreatic cancer. EUS: endoscopic ultrasound

Modalities	Advantage	Disadvantage
EUS	High resolution, can get tissue biopsy	Invasive
CT scan	Provides detailed imaging, easily available	Can miss diagnosis sometimes
MRI	Can detect small tumors that might be missed by other scans	Expensive; not widely available

Artificial intelligence in radiology

The use of AI in the diagnosis of cancer can help physicians save a lot of time by manually going over the details of imaging in detail. One method to extract minute details from the detailed image that might have been missed by the human eye is the use of radiomics. This method is used to extract information regarding shape, characteristics, heterogeneity, and even tumor aggressiveness [13].

AI models are being made that can help analyze CT scan images in more detail and provide a more detailed analysis of complex patterns and images [14]. AI techniques have also been used to predict the malignant potential of intraductal mucinous papillary neoplasm (IPMN) using CT scan images [15]. In one of the studies, a computational model integrating clinical data and imaging features was designed that was used to predict the probability of lymph node metastasis in PDAC by integrating the clinical data and the images extracted from CT scans [16].

Endoscopic ultrasound is also used to detect small tumors and lesions of the pancreas up to a range of 2-3 mm in size [17]. It can help to get detailed images of the pancreas and surrounding tissue. In one of the studies carried out by Zu et al, a machine model was used to extract EUS images recorded for pancreatic adenocarcinoma and chronic pancreatitis. The model then helped to detect features that were used to differentiate between PC and chronic pancreatitis with a sensitivity of 94% [18].

PET scan is also used in staging pancreatic cancer and monitoring response to treatment, as well as looking for recurrent disease. Zhang used PET/CT scan imaging and developed a machine model that is used to differentiate between autoimmune pancreatitis and pancreatic adenocarcinoma non-invasively [19].

Digital pathology is another method that can be used to analyze histological slides with accuracy and precision that exceeds that of a human. This involves the utilization of scanners which are used to scan a simple tissue or blood specimen and form an accurate 3D image of the entire tissue. In addition to diagnosing biopsy samples, this technology can help us better understand the cellular structure [20].

Models have also been made that can detect tumor markers or protein-based markers that are present more in cancer cells than in normal cells. Early work shows that AI models such as surface-enhanced laser desorption/ionization (SELDI) have been used to detect six protein markers that are found in cancer cells and not detected in normal pancreatic cells [21]. Building on the utilization of biomarkers, AI can also analyze genomic data and find associations of specific genes to pancreatic cancer. Ko et al developed a machine model, Genes Vector for Each Sample (GVES), that can be used to diagnose genes involved in the prognosis of the disease with accuracy, irrespective of datasets and sample sizes [22].

AI is also used to predict the risk of the development of pancreatic cancer by extracting clinical data about the patient from electronic health systems [23]. A few studies have been done that show that the use of electronic health records (EHR) to extract patient data can screen people who are at increased risk of developing pancreatic cancer before diagnosis [24]. In one article, logistic regression was used on EHR to detect people at risk of cancer a decade before the diagnosis was made [24].

The use of AI and software can help physicians analyze images and tissue samples to detect pancreatic cancer, predict grading and staging, and detect people who might develop PCs in the future.

Artificial intelligence use in the treatment of pancreatic cancer

The use of AI in the treatment of pancreatic cancer has the potential to improve patient outcomes and accelerate the development of new treatments. By providing personalized treatment plans, identifying targeted therapies, monitoring treatment response, optimizing radiation therapy, and matching patients to clinical trials, AI can help physicians provide the best possible care for pancreatic cancer patients.

Personalized Treatment Plans

AI can analyze various types of data, including medical history, imaging results, and genetic data to create personalized treatment plans [23-25]. AI can use ML algorithms to analyze CT scans, MRI scans, and other imaging data to create a 3D model of the tumor and surrounding tissues. This can help physicians plan intervention and radiation therapy with greater accuracy while minimizing the risk of complications. AI can also use genetic data to identify specific mutations or molecular targets that are present in cancer cells but not in normal cells [26,27]. This can help physicians develop personalized treatment plans that are tailored to the unique characteristics of each patient's cancer.

Targeted Therapies

AI can be used to identify specific molecular targets that are essential for the growth and survival of pancreatic cancer cells [28]. This information can be used to develop targeted therapies that specifically inhibit these targets. ML algorithms can analyze genetic data to identify specific mutations that are present in cancer cells but not in normal cells [29]. This can be used to develop drugs that specifically target those mutations, which can be more effective and less toxic than traditional chemotherapy. AI can also be used to predict which patients are most likely to respond to specific treatments, enabling physicians to personalize treatment plans based on the patient's genetic profile.

Treatment Monitoring

AI can be used to monitor a patient's response to treatment and adjust the treatment plan accordingly [30]. For example, ML algorithms can analyze imaging data to track changes in tumor size over time [31]. This can help physicians determine whether the treatment is working or if adjustments need to be made. AI can also be used to monitor a patient's blood markers, such as CA 19-9, which can indicate the presence of pancreatic cancer [32]. By monitoring treatment response, physicians can adjust treatment approaches to maximize the chances of a successful outcome.

Radiation Therapy

AI can also be used to optimize radiation therapy for pancreatic cancer patients. This can include creating a personalized radiation treatment plan based on the patient's medical history, imaging results, and other data [33]. ML algorithms can analyze imaging data to identify areas of the tumor that require higher doses of radiation. This can help physicians deliver more effective radiation therapy while minimizing the risk of damage to healthy tissue. AI can also be used to monitor the patient's response to radiation therapy and adjust the treatment plan accordingly [31].

Clinical Trial Matching

Matching patients to clinical trials based on their medical history and other criteria is also an application of AI [34,35]. This can help speed up the drug development process and improve patient outcomes. ML algorithms can analyze genetic data to identify patients who are likely to respond to a particular treatment. This can help identify patients who are eligible for a clinical trial and improve the chances of success for the trial.

Table 2 presents a comparison of various AI models that have been used in studies for predicting the risk and outcomes in pancreatic cancer.

**Table 2:** Comparison of various AI models used in studies for predicting risk and outcomes in pancreatic cancer.

Study	AI model used	Input data used	Outcomes Predicted	Performance	Limitations
Malhotra et al. [24]	Multivariate Logistic regression (MLR), Random Forest (RF)	Smoking, Alcohol intake, Weight and BMI, symptoms, Past Medical History, Medication History, Primary care consultation frequency	Screening tool predicting risk of PC development	72.5% sensitivity, 59% specificity for patients <60 y; 65% sensitivity, 57% specificity for patients >60 y.	High number of false positives; poor external validity
Roch et al. [25]	Natural Language Processing (NLP)- based pancreatic cyst identification system	Pancreatic cyst occurrence from electronic health records	Pancreatic cyst identification; subsequent follow-up for patients at risk of developing PC	99.9% sensitivity, 98.8% specificity.	Data from only one medical center used; no other risk factors taken into consideration
Almeida et al. [26]	Artificial Neural Network (ANN)	Differentially expressed genes identified as risk factors for pancreatic cancer (PC)	Identified 5 biomarkers for pancreatic ductal adenocarcinoma (PDAC) based on performance (FAIM3, IRANK3, DENND2D, PLBD1, AGPAT)	100% sensitivity, 94% specificity.	Variations in sample preparation or microarray technologies.
Wang, Liu, Ma et al. [27]	Support Vector Machine Recursive Feature Elimination (SVM-RFE) and Large Margin Distribution Machine Recursive Feature Elimination (LDM-RFE)	Differentially expressed genes identified as risk factors for PC	Three genes (MMP7, FOS and A2M) were found to be closely related to the survival rate of the patients.	80% Specificity	Database-based population - not self-measure

Pancreatic cancer overall survival prediction

Pancreatic cancer usually has a very poor prognosis of a 5-year survival rate of less than 6%. Determining the survival rate of pancreatic cancer is hard as the tumor usually presents with different features. Research is being done to predict the survival time of pancreatic cancer using machine models that are non-invasive and accurate. CT and PET scan images have been used with machine models to predict the survival time of PC patients with more accuracy [36]. AI has also been used to predict the survival time when imaging is not available. In one of the studies carried out by Hayward et al, they used clinical features and treatment records to predict the outcomes and survival time [37]. Biomarkers and genes have also been used to determine prognosis via ML. Algorithms have been used to analyze the identified biomarkers and genes, which has helped in developing prognostic classifiers [38].

AI has revolutionized the medical field and made work easier for physicians. For pancreatic cancer, the application of this technology is wide-ranging, from early diagnosis to predicting survival rates. Many models have been designed that can help in predicting the recurrence of pancreatic cancer after treatment [39].

Future perspectives

AI prediction power will improve by training deep learning models with more input data. As pancreatic cancer is prevalent worldwide, multi-institutional collaboration could help create a larger cohort of patients for evaluation. These studies should focus on factors related to the prognosis and treatment of pancreatic cancer (such as identifying ambiguous pancreatic lesions, the presence of vascular invasion, and the treatment response). Another significant aspect is the utilization of AI for the analysis of PDAC behavior in patients with risk factors for PDAC vs normal patients. Further study is also needed to demonstrate the efficacy of AI for medical assistance by comparing the output of medical staff using AI aid to that of experts not using AI support. The perception and acceptance of AI by healthcare providers and patients also determine the extent of its use in the future. AI knowledge among healthcare providers has a greater impact on their readiness to learn and apply its technology in their field, especially in the area of pancreatic cancer. Gaining patient trust through education also determines the future success of AI in clinical practice.

Limitations

In the area of medicine, AI has its limitations. Even though AI has potential use for pancreatic cancer diagnosis and treatment, it may not be ready for practical usage without further refinement. The truthfulness of AI can be influenced by irrelevant databases with biases.

Therefore, it is crucial to design a non-biased, multicenter collaborative study, taking also into consideration other important aspects, such as economics, ethical evaluation, and medical professional regulations.

Ethical considerations

The advent of AI in pancreatic carcinoma diagnosis and treatment presents critical ethical concerns. Protecting patient data for AI training while ensuring privacy remains paramount, necessitating stringent data privacy measures. AI should augment healthcare professionals, not replace them, emphasizing transparency in AI's capabilities and limitations. Mitigating biases within AI algorithms is crucial to preventing disparities in diagnosis and treatment. Equitable access to AI-driven healthcare services and addressing resource disparities are imperative ethical goals. Continuous discussions and regulations are essential to govern AI's ethical implementation and impact on patient care, requiring collaboration among regulators, healthcare professionals, technologists, and ethicists to establish ethical frameworks prioritizing patient well-being in this evolving landscape.

## Conclusions

AI has the potential to revolutionize the diagnosis and treatment of pancreatic cancer, which is a highly lethal disease with a low survival rate. Early detection and accurate diagnosis are critical for improving patient outcomes, and AI can analyze large amounts of patient data and medical images to identify patterns and relationships that can lead to earlier and more accurate diagnoses. By analyzing genetic profiles, imaging studies, and electronic health records, AI can identify specific biomarkers or genetic mutations that are associated with pancreatic cancer, which can aid in early detection and diagnosis. Additionally, AI can assist in the staging of pancreatic cancer by analyzing radiographic data to determine the extent of the disease, which can guide treatment decisions.

In terms of treatment, pancreatic cancer is often resistant to chemotherapy and radiation therapy, which makes it difficult to effectively treat. However, AI can help to identify more effective treatment by simultaneously analyzing a wide variety of factors such as genetic profiles, treatment histories, and clinical outcomes. This can lead to the development of more personalized and effective treatment plans, as well as improve patient survival rates. AI can also assist in the drug discovery process by identifying new therapeutic targets and predicting the efficacy of potential drugs.

Overall, the use of AI in pancreatic cancer is an area of active research and development that has the potential to transform patient care and management in the field of oncology. By providing more objective and consistent assessments of medical images and analyzing large amounts of patient data, AI has the potential to improve early detection, increase diagnostic accuracy, and guide treatment decisions, ultimately leading to better patient outcomes and more efficient use of healthcare resources. However, more research is needed to fully realize the potential of AI in the field of pancreatic cancer.
